# Low rather than high interleukin-6 levels are associated with immune-related adverse events in cancer patients treated with immune checkpoint inhibitors

**DOI:** 10.3389/fimmu.2025.1677778

**Published:** 2025-09-12

**Authors:** Iñigo Les, David de Haedo, Mireia Martínez, Berta Ibáñez-Beroiz, Amaia Moreno, Ibone de Elejoste, Ana Campillo-Calatayud, Inés Pérez-Francisco, María Cabero, Iñaki Elejalde, Virginia Arrazubi

**Affiliations:** ^1^ Servicio de Medicina Interna, Hospital Universitario de Navarra, Pamplona, Spain; ^2^ Unidad de Enfermedades Autoinmunes, Servicio de Medicina Interna, Hospital Universitario de Navarra, Pamplona, Spain; ^3^ Grupo de Enfermedades Inflamatorias e Inmunomediadas, Navarrabiomed-Universidad Pública de Navarra, Instituto de Investigación Sanitaria de Navarra (IdISNA), Pamplona, Spain; ^4^ Servicio de Oncología Médica, Hospital Universitario Araba, Osakidetza-Servicio Vasco de Salud, Vitoria-Gasteiz, Spain; ^5^ Instituto de Investigación Sanitaria Bioaraba, Grupo de Investigación en Cáncer de Pulmón, Vitoria-Gasteiz, Spain; ^6^ Servicio de Metodología, Navarrabiomed-Universidad Pública de Navarra, IdISNA, Pamplona, Spain; ^7^ Red de Investigación en Cronicidad, Atención Primaria y Promoción de la Salud (RICAPPS), Spain; ^8^ Servicio de Oncología Médica, Hospital Universitario Galdakao, Galdácano, Spain; ^9^ Servicio de Oncología Médica, Hospital Universitario Donostia, San Sebastián, Spain; ^10^ Instituto de Investigación Sanitaria Bioaraba, Grupo de Investigación en Cáncer de Mama, Vitoria-Gasteiz, Spain; ^11^ Instituto de Investigación Sanitaria Bioaraba, Plataforma de Ensayos Clínicos, Vitoria-Gasteiz, Spain; ^12^ Servicio de Oncología Médica, Hospital Universitario de Navarra, Instituto de Investigación Sanitaria de Navarra (IdISNA), Pamplona, Spain

**Keywords:** immune checkpoint inhibitors, immune-related adverse events, cancer, interleukin-6, sex, immune-mediated diseases

## Abstract

**Background:**

Among the biomarkers associated with immune-related adverse events (irAEs) induced by immune checkpoint inhibitors (ICIs) in cancer patients, interleukin-6 (IL-6) has emerged as a key predictive factor. However, it remains unclear whether high or low levels of IL-6 predispose patients to irAEs. Our objective was to evaluate the strength of the association between circulating IL-6 levels, measured in cancer patients before and after initiating ICIs, and the risk of irAEs.

**Methods:**

In this multicenter, prospective pan-cancer cohort study, serum IL-6 levels were quantified immediately before the first (pre-ICI) and second (post-ICI) cycles of ICI therapy. To assess the association between IL-6 and irAEs, Fine and Gray competing risk regression models were fitted, considering irAEs as the main event and death as the competing event. The incremental predictive value of IL-6 levels was evaluated using Harrell’s C-index.

**Results:**

Overall, 224 patients were followed up for a median of 75.5 days after ICI initiation. The adjusted 1-year cumulative incidence of irAEs was 49.0% (95% confidence interval [95%CI], 41.9-55.6%). Multivariate regression models identified female sex (hazard ratio [HR], 1.81; 95%CI, 1.17-2.81; p=0.008), dual ICI therapy with nivolumab plus ipilimumab (HR, 1.86; 95%CI, 1.14-3.02; p=0.012) and post-ICI IL-6 levels (HR, 0.97; 95%CI, 0.94-1.00; p=0.049) as independent risk factors for irAEs. Using standardized post-ICI IL-6 levels, the effect was stronger, with an HR of 0.74 (95% CI, 0.55-1.00; p=0.049). Adding post-ICI IL-6 levels to a model containing established irAE risk factors improved the Harrell’s C-index from 0.623 to 0.640.

**Conclusion:**

In cancer patients treated with ICIs, low rather than high post-ICI IL-6 levels, female sex and dual ICI therapy are independent risk factors for irAEs.

## 1 Introduction

Immune-related adverse events (irAEs) represent a distinct category of drug toxicity that affects as many as half of cancer patients receiving immune checkpoint inhibitor (ICI) therapy within the first year of treatment (1). The development of irAEs has a substantial impact on the quality of life, prognosis and healthcare costs of patients with cancer (2–4). Paradoxically, the occurrence of irAEs has been associated with longer overall survival rates (5), suggesting that maintaining ICI therapy could benefit patients who experience immune-related toxicity, provided this toxicity is not life-threatening. Therefore, identifying predictive factors of irAEs is crucial to optimize clinical monitoring and improve patient adherence to ICI therapy (6).

Among the various biomarkers associated with irAEs, interleukin-6 (IL-6) has gained increasing prominence (7, 8). IL-6 is a pleiotropic proinflammatory cytokine involved in the pathogenesis of several immune-related diseases, including irAEs, as well as tumorigenesis (9). Its oncogenic effect has been linked to IL-6-induced transcription of intermediary molecular factors that drive cell cycle progression, angiogenesis, tumor invasiveness and metastasis (10). Indeed, many studies have associated the presence of high plasma levels of IL-6 —both before and after ICI administration— with an impaired antitumor response to ICIs, poorer clinical outcomes and increased risk of irAE development (11–14). Conversely, other authors have suggested that low plasma levels of IL-6 may correlate with the occurrence of irAEs (15), as IL-6 is known to play a role in promoting immune evasion by cancer cells (16, 17). Therefore, it remains unclear whether high or low levels of IL-6 predispose patients receiving ICI therapy to irAEs. Addressing this question is clinically relevant, as a potential association between elevated IL-6 levels and irAEs could justify the pre-emptive use of IL-6 blocking agents to reduce both the incidence and severity of irAEs (18, 19). Indeed, given the previously proposed decoupled effect of IL-6 blockade in enhancing both anti-tumor and anti-inflammatory responses, several clinical trials have been initiated in recent years to evaluate the efficacy and safety of IL-6-targeting therapies in patients undergoing ICI treatment (20). The results of these ongoing clinical trials are awaited.

To date, few studies have prospectively assessed the association between circulating IL-6 levels and the risk of developing irAEs. Most research in this field has focused on measuring IL-6 levels at the time of irAE detection rather than before the onset of ICI-induced toxicities (21). Moreover, the potential diagnostic value of sequential IL-6 measurements after ICI initiation for predicting irAEs remains to be clarified. In addition, in the current landscape of expanding approvals of ICI therapies and the corresponding rise in irAE incidence worldwide, female sex has emerged as an independent risk factor for immune-related toxicities (22). Notably, a recent study by our group found a significant interaction between female sex and another predictor of irAEs, namely, absolute neutrophil count before the first ICI cycle (23). Similarly, previous data suggest that women are at a higher risk of developing irAEs than men at equivalent peripheral IL-6 levels, pointing to a sex-dependent IL-6-mediated mechanism underlying ICI-induced toxicities (15). In line with these findings, in other inflammatory conditions, pathogenic IL-6 levels associated with disease onset, progression and complications also differ between sexes (24, 25).

Based on the available evidence, we hypothesized that baseline and follow-up circulating IL-6 levels might be associated with irAE incidence. Furthermore, we anticipated differences in irAE risk depending on sex and other patient characteristics. Therefore, the main objective of this study was to examine the impact of IL-6 levels measured in the peripheral blood of cancer patients before and after ICI initiation on the risk of developing irAEs. In addition, subgroup analyses were planned to assess the association between IL-6 levels and irAEs depending on clinically relevant variables —including age, sex, tumor origin and comorbidities— in a pan-cancer cohort of ICI-treated patients.

## 2 Materials and methods

### 2.1 Study design and ethics statement

This study used preliminary data from the AUTENTIC project, described in detail elsewhere (26). AUTENTIC is a multicenter prospective observational pan-cancer cohort designed to identify biomarkers potentially predictive of irAEs in patients receiving ICIs for solid tumors. Between February 2021 and March 2024, patients were consecutively enrolled by medical oncologists at outpatient clinics in four tertiary hospitals in northern Spain. All participants provided written informed consent prior to inclusion in the study.

The study was conducted in accordance with the International Council for Harmonization Guidelines for Good Clinical Practice version E6(R2) (27), the principles of the Declaration of Helsinki and local regulations. Ethical and regulatory approval was obtained from the Spanish Agency of Medicines and Medical Devices (code: ILB-NIV-2018-01), the Basque Country Research Ethics Committee (code: PI2018106 [EPA-SP]) and local ethics committees of each participating hospital. The study protocol is publicly registered on ClinicalTrials.gov (identifier: NCT03868046).

### 2.2 Study population

Eligible participants were required to fulfil the following inclusion criteria: (1) initiation of treatment with a single ICI or dual ICI therapy in accordance with current clinical guidelines; (2) ICI-naïve status, although prior systemic cancer therapies, such as chemotherapy or tyrosine kinase inhibitors, were permitted; and (3) provision of written informed consent. Exclusion criteria were: (1) an estimated life expectancy of less than 3 months from the initiation of ICI therapy; (2) any contraindication to ICIs, including active severe autoimmune diseases or an Eastern Cooperative Oncology Group (ECOG) performance status ≥3; (3) concurrent use of chemotherapy, tyrosine kinase inhibitors or other targeted anti-cancer agents; or (4) ongoing immunosuppressive therapy, including systemic glucocorticoids at doses exceeding 10 mg/day of prednisone or equivalent.

### 2.3 Procedures

Once enrolled in the study, patients were managed according to current clinical practice guidelines on solid cancer immunotherapy and related toxicities (28). Clinical visits and follow-up intervals were scheduled based on each specific ICI regimen, occurrence of irAEs or other complications, and the discretion of the attending physician. The study was prospectively monitored from initiation to final follow-up, with all irAEs documented by treating physicians and independently verified by the monitoring team. Agents administered in this study included anti-programmed cell death protein-1 (PD-1) antibodies (pembrolizumab, nivolumab, cemiplimab and dostarlimab), anti-PD-1 ligand 1 (PD-L1) antibodies (atezolizumab, durvalumab and avelumab) and an anti-cytotoxic T-lymphocyte-associated antigen-4 (CTLA-4) antibody (ipilimumab). Dual ICI therapy was defined as the co-administration of ipilimumab with a PD-1 inhibitor, specifically nivolumab. Two blood samples were required: a baseline sample and a follow-up sample, collected immediately before the first and second ICI cycles (hereafter, referred to as the ‘pre-ICI’ and ‘post-ICI’ samples, respectively). In the absence of complications that could delay dosing, the interval between the two samples was 2–3 weeks, in accordance with the product-specific dosing intervals outlined in regulatory summaries. Blood samples were processed applying a standardized serum collection protocol, and IL-6 levels in both pre- and post-ICI samples were quantified using the Human IL-6 Enzyme Linked Immunosorbent Assay (ELISA) Kit by ThermoFisher^®^, according to the manufacturer’s instructions.

### 2.4 Outcome

The primary endpoint was defined as the cumulative incidence of the first irAE of any grade during the follow-up period. An irAE was defined as any symptom, sign, syndrome or disease resulting from an immune-mediated mechanism during the follow-up of ICI therapy once other causes, such as infectious diseases and cancer progression, had been ruled out. Grades and types of irAEs were categorized according to the Common Terminology Criteria for Adverse Events version 5.0 (29).

### 2.5 Variables

#### Exposure and confounding variables

Consistent with the study hypothesis, we generated a directed acyclic graph (DAG) diagram representing the pathogenic role of potential explanatory variables involved in the development of irAEs (**Supplementary Figure S1**). Based on this DAG, we evaluated pre- and post-ICI IL-6 levels as exposure variables for irAEs. Other potential irAE risk factors, namely, patient age, sex, pre-existing immune-mediated disease and dual ICI therapy, were considered confounders. Patient age was calculated from the date of birth to the date of ICI initiation. The diagnosis of pre-existing immune-mediated diseases in patients included in the study was based on current international consensus classification criteria, verified through a review of electronic health records and confirmed by the patient’s referring oncologist. Dual ICI therapy was considered to be a variable of interest based on previous data from a meta-analysis, which reported an irAE rate of up to 90% when ipilimumab was combined with nivolumab (1).

For exploratory purposes only, we also evaluated whether the appearance of irAEs was influenced by other variables, such as primary tumor origin, smoking and alcohol consumption, performance status, body mass index (BMI), comorbidities, glomerular filtration rate, (neo)adjuvant intent of ICI therapy and circulating blood cell counts and ratios.

#### Subgroup-defining variables

We planned to perform multiple prespecified subgroup analyses as a function of the following variables and categories, using predefined cut-off values for quantitative variables derived from previously published data, as indicated in parentheses: sex (female, male), primary tumor origin (lung cancer, non-lung cancer), pre-ICI absolute lymphocyte peripheral count (>2.0 thousand cells per microliter [K/µl], ≤2.0 K/µl) (23), post-ICI absolute neutrophil peripheral count (>4.0 K/µl, ≤4.0 K/µl) (23), smoking status (never, former or current smoker), BMI (≥25 kg/m^2^, <25 kg/m^2^) (30), performance status (ECOG ≤1, ECOG =2) (31) and comorbidities assessed using the abbreviated version of the Charlson comorbidity index (aCharlson =2, aCharlson ≥3) (32). In the aCharlson index, one point is assigned for cardiovascular disease, diabetes, heart failure, chronic obstructive pulmonary disease, dementia and peripheral artery disease, and two points are added for chronic renal failure and cancer. In our cohort, all patients had cancer, and therefore, the aCharlson index ranged from 2 to 10 points.

### 2.6 Sample size and statistical analysis

For comparative purposes, IL-6 levels were standardized by subtracting the mean from the raw value and dividing by the standard deviation. Assuming a significance level of α=0.05, an irAE probability of π=0.45 during the follow-up and a correlation with the rest of variables of ρ=0.05, the available sample size (n=224) provided a power equal to 80% to detect as significant hazard ratios (HRs) of a magnitude equal to 0.75 for the exposure variables (standardized IL-6 levels, variance σ^2^ = 1). This sample size calculation was performed with the powerSurvEpi package in R software.

To describe the baseline characteristics and treatment data of the cohort, quantitative variables were reported as means with standard deviations or medians with ranges, as appropriate. Categorical variables were expressed as frequencies with percentages. Comparisons of IL-6 levels by sociodemographic and clinical variables were performed using the Mann-Whitney U test for unpaired data, while changes in IL-6 levels between before and after the first ICI cycle were assessed with the Wilcoxon signed-rank test for paired data and represented using a violin plot.

The cumulative irAE incidence over time was analyzed and plotted using Fine and Gray competing risk survival analysis, considering irAEs as the main event, death as the competing event and both loss to follow-up and cancer progression as right-censored observations. To assess the association between IL-6 levels and irAEs, univariate Fine and Gray competing risk regression models were fitted considering pre- and post-ICI IL-6 levels as exposure variables, irAEs as the main event and death as the competing event. Based on the study DAG diagram (**Supplementary Figure S1**), patient age, sex, pre-existing immune-mediated disease before ICI initiation and dual ICI therapy were also included in the model as confounding variables. A multivariate Fine and Gray competing risk regression model was fitted to determine the independent contribution of each factor while adjusting for the others. In this model, quantitative pre- and post-ICI IL-6 levels were analyzed using both raw and standardized values to estimate HRs per one standard deviation in IL-6. Results were expressed as HRs and 95% confidence intervals (CIs).

For subgroup analyses, we re-ran the Fine and Gray multivariate regression models, stratifying the cohort by sex (female, male), primary tumor origin (lung cancer, non-lung cancer), pre-ICI absolute lymphocyte peripheral count (>2.0 K/µ, ≤2.0 K/µl), post-ICI absolute neutrophil peripheral count (>4.0 K/µl, ≤4.0 K/µl), smoking status (never smoker, former or current smoker), BMI (≥25 kg/m^2^, <25 kg/m^2^), performance status (ECOG ≤1, ECOG =2) and comorbidity index (aCharlson=2, aCharlson≥3). For illustrative purposes, subgroup analyses were performed using standardized IL-6 levels and presented as a forest plot. Finally, the incremental predictive value of IL-6 levels relative to a model including other explanatory variables was evaluated by comparing Harrell’s C-index of the two models (with and without IL-6 levels), adjusted for overfitting by bootstrap resampling.

Statistical analyses were conducted using R software (version 4.4.0) for Windows^®^.

## 3 Results

### 3.1 Description of the cohort

Baseline characteristics of the patients and the ICIs prescribed are summarized in **Table 1**. A total of 224 patients were consecutively included in the study and followed up for a median of 75.5 (range, 6-949) days from the time of ICI initiation. The mean age of the cohort was 66.9 ± 9.4 years, and three-quarters of the patients were men. The most frequent primary malignancy was non-small cell lung cancer (diagnosed in 84 patients, 37.5%). A history of immune-mediated disease before starting ICI therapy was documented in 17 patients (7.6%), the most common being psoriasis (8 cases); none showed clinical evidence of disease activity at inclusion. Overall, 104 patients (46.4%) had a comorbidity other than cancer, as assessed by an aCharlson index >2, at the time of ICI initiation. At baseline, only one patient was on prednisone, and the dose received was <10 mg/day in compliance with the study entry criteria.

**Table 1:** Baseline characteristics and therapy-related factors in the cohort studied (n = 224).

Variable	Statistic
Age at ICI initiation, *mean (SD)*	66.9 (9.4)
Sex, *n (%)*	
Male	168 (75.0)
Female	56 (25.0)
ECOG score, *n (%)*	
0	65 (29.0)
1	134 (59.8)
2	25 (11.2)
Smoking history, *n (%)*	
Never smoker	46 (20.5)
Current or former smoker	178 (79.5)
Body mass index*, *mean (SD)*	26.0 (4.6)
Renal failure^†^, *n (%)*	45 (20.1)
Immune-mediated disease, *n (%)*	17 (7.6)
Psoriasis	8 (3.6)
Thyroiditis	2 (0.9)
Others	7 (3.1)
Systemic lupus erythematosus	1
Multiple sclerosis	1
Autoimmune haemolytic anaemia	1
Ankylosing spondylitis	1
Membranous glomerulonephritis	1
Interstitial pneumonia with autoimmune features	1
Vitiligo	1
Abbreviated Charlson index, *n* (%)	
2	120 (53.6)
3	61 (27.2)
4 or higher	43 (19.2)
Primary cancer, *n (%)*	
Non-small cell lung cancer	84 (37.5)
Renal cell carcinoma	40 (17.9)
Urothelial carcinoma	33 (14.7)
Head and neck squamous carcinoma	29 (12.9)
Melanoma	19 (8.5)
Gastric adenocarcinoma	6 (2.7)
Colorectal adenocarcinoma	5 (2.2)
Others	8 (3.6)
Esophageal carcinoma^‡^	2
Malignant pleural mesothelioma	2
Pancreatic adenocarcinoma	1
Endometrial adenocarcinoma	1
Cutaneous squamous-cell carcinoma	1
Merkel cell carcinoma	1
Treatment line at ICI initiation, *n* (%)	
Adjuvant	29 (12.9)
Non-adjuvant	195 (87.1)
First-line	119
Second-line	68
Third-line and beyond	8
Treatment regimen, *n (%)*	
Monotherapy	192 (85.7)
Pembrolizumab	60 (26.8)
Nivolumab	44 (19.6)
Atezolizumab	30 (13.4)
Durvalumab	28 (12.5)
Avelumab	16 (7.1)
Cemiplimab	12 (5.4)
Dostarlimab	2 (0.9)
Dual therapy (Ipilimumab plus Nivolumab)	32 (14.3)

ECOG, Eastern Cooperative Oncology Group; ICI, immune checkpoint inhibitor; n, number; SD, standard deviation.

* Calculated as: body mass index = weight (kg) / height (m)^2^.

^†^ Defined as a baseline glomerular filtration rate below 60 mL/min/m^2^.

^‡^ Including one case each of esophageal adenocarcinoma and esophageal squamous-cell carcinoma.

Pre- and post-ICI samples were available for 224 (100%) and 207 (92.4%) patients, respectively. The reasons for the 17 missing post-ICI samples were death (n=5), cancer progression (n=8) and irAE diagnosis (n=2), all occurring before the second ICI cycle, as well as sample loss (n=2). Patients with baseline age ≥70 years, ECOG >0, primary lung cancer, aCharlson index >2 and/or receiving non-adjuvant ICI therapy had significantly higher pre-ICI IL-6 levels. In contrast, women and patients with pre-existing immune-mediated diseases showed a trend towards lower pre-ICI IL-6 levels (**Supplementary Table S1**). Median IL-6 levels did not change significantly between pre- and post-ICI samples: 2.0 pg/ml (range, 0-133.9) vs. 2.2 pg/ml (range, 0-102.1) pg/ml; p=0.780 (**Supplementary Figure S2**).

### 3.2 Outcomes

Accounting for death as a competing risk, the 1-year cumulative incidence of a first irAE was 49.0% (95% CI, 41.9-55.6%) (**Supplementary Figure S3**). The median time to irAE onset was 46 (range, 10-634) days from the time of ICI initiation, which coincided with the pre-ICI sample collection, and 25 (range, 0-615) days from the post-ICI sample collection. During the entire follow-up period, 105 patients (46.9%) experienced at least one irAE. Of these 105 first irAEs, 47 (44.8%) were categorized as grade 1, 52 (49.5%) as grade 2 and 6 (5.7%) as grade 3. The most common types of first irAE were cutaneous and endocrinological, accounting for 36 cases (34.6%) and 27 cases (26.0%), respectively (**Table 2**).

**Table 2:** Summary of the first immune-related adverse events in patients in the cohort*.

irAE type	*n* (%)
Dermatologic	36 (34.3)
Maculopapular rash	21 (20.0)
Psoriasis	5 (4.8)
Pruritus	4 (3.8)
Other cutaneous irAEs**^†^**	6 (5.7)
Endocrinological	27 (25.8)
Hypothyroidism	12 (11.4)
Hyperthyroidism	10 (9.5)
Thyroiditis	1 (1.0)
Hypophysitis	2 (1.9)
Adrenal insufficiency	1 (1.0)
Hyperandrogenism	1 (1.0)
Musculoskeletal	6 (5.7)
Inflammatory arthritis	3 (2.8)
Arthromyalgia	2 (1.9)
Polymyalgia rheumatica	1 (1.0)
Enterocolitis	15 (14.3)
Pneumonitis	8 (7.6)
Nephritis	5 (4.8)
Hepatitis	3 (2.8)
Hematological irAEs^‡^	3 (2.8)
Miscellaneous^¶^	2 (1.9)
Overall	105 (100.0)

irAE, immune-related adverse event; n, number.

* Only the first irAE occurring in patients who experienced more than one irAE was included in the analysis.

**^†^** Including three cases of mucositis, two cases of xerosis cutis and one case of lichen planus.

^‡^ Including one case each of hemolytic anemia, neutropenia and pancytopenia (all recovering during follow-up).

^¶^ Including one case each of systemic lupus erythematosus flare and uveitis.

Patients who developed irAEs showed a trend towards lower IL-6 levels than those who did not, both in pre-ICI (1.7 pg/ml [range, 0-112.2] vs. 2.5 pg/ml [range, 0-133.9]; p=0.054) and post-ICI (1.9 pg/ml [range, 0-25.9] vs. 2.9 pg/ml [range, 0-102.1]; p=0.094) samples.

### 3.3 Factors associated with immune-related adverse events

**Figure 1:**
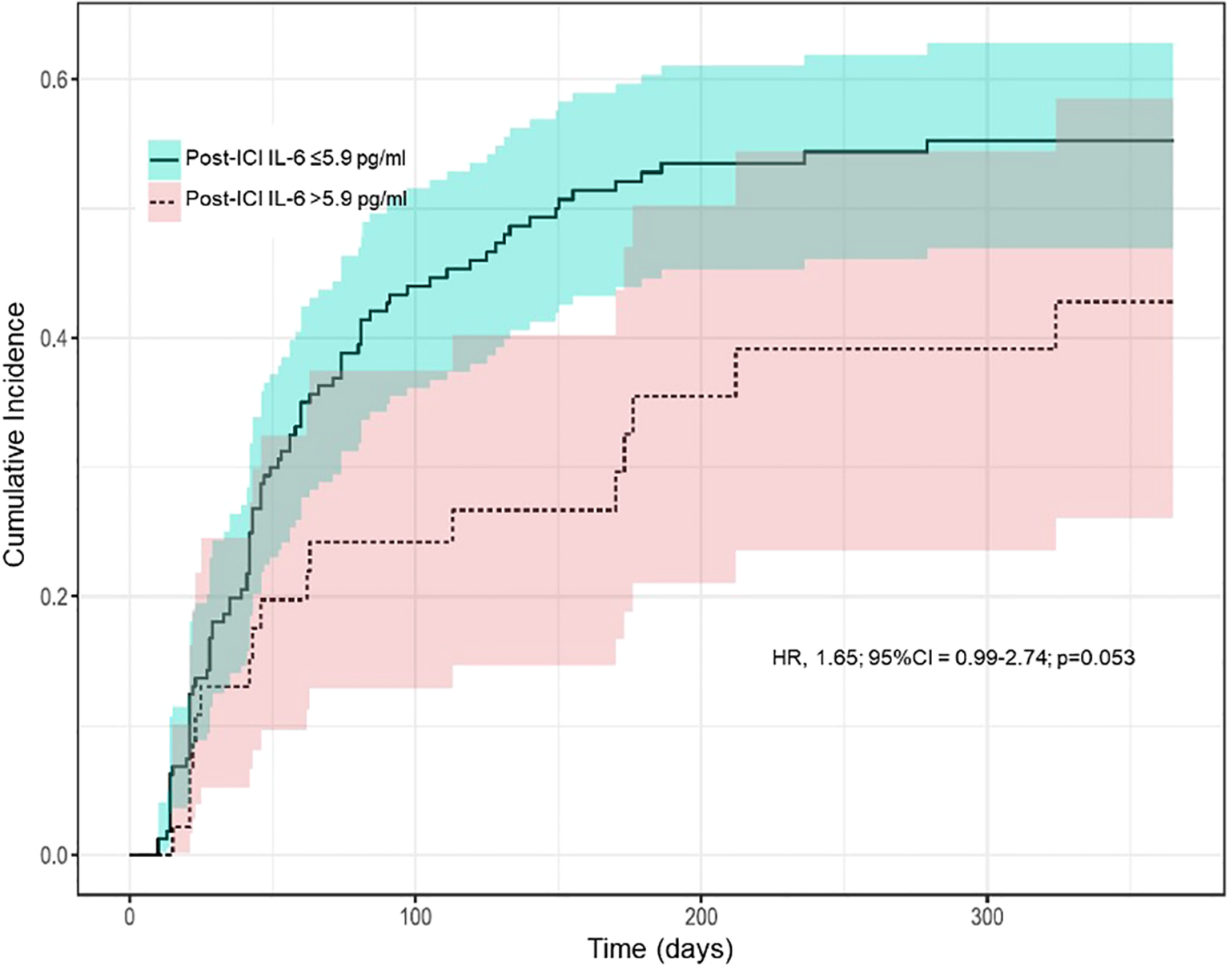
Cumulative incidence of immune-related adverse events over time by predefined cut-off values of interleukin-6 levels measured immediately before the second immune checkpoint inhibitor cycle. Abbreviations (in alphabetical order): CI, confidence interval; HR, hazard ratio; ICI, immune checkpoint inhibitor; IL-6, interleukin-6. The plot was generated using the Fine and Gray competing risk survival analysis, considering immune-related adverse events as the main event and death as the competing event. The cut-off of 5.9 pg/ml was chosen based on data published previously by Valpione et al. (reference 15).

In the univariate Fine and Gray competing risk regression models, variables associated with irAEs were female sex (HR, 1.73; 95% CI, 1.12-2.67; p=0.013), pre-existing immune-mediated disease before ICI initiation (HR, 1.84; 95% CI, 1.19-2.85; p=0.007), dual ICI therapy (HR, 1.97; 95% CI, 1.24-3.13; p=0.004) and low post-ICI IL-6 levels (HR, 0.96; 95% CI, 0.93-0.99; p=0.015). In contrast, other potentially explanatory variables, such as age and pre-ICI IL-6 levels, did not reach statistical significance in our analysis (**Table 3**). Using predefined IL-6 cut-off values (15), the cumulative incidence of irAEs was higher in patients with post-ICI IL-6 levels ≤5.9 pg/ml than those with post-ICI IL-6 levels >5.9 pg/ml (**Figure 1**).

**Table 3:** Factors associated with a first immune-related adverse event in the cohort.

Univariate analysis			
Variable	HR	95% CI	*p*-value
Patient age at inclusion	1.00	0.98-1.02	0.99
Patient sex (female)	1.73	1.12-2.67	0.013
Immune-mediated disease before ICI therapy	1.84	1.19-2.85	0.007
Dual ICI therapy*	1.97	1.12-2.67	0.013
Pre-ICI IL-6 levels	0.99	0.97-1.01	0.29
Post-ICI IL-6 levels	0.96	0.93-0.99	0.015
Multivariate analysis			
Variable	HR	95% CI	*p*-value
Patient age at inclusion	1.01	0.99-1.03	0.530
Patient sex (female)	1.81	1.17-2.81	0.008
Immune-mediated disease before ICI therapy	1.49	0.90-2.46	0.120
Dual ICI therapy*	1.86	1.14-3.02	0.012
Post-ICI IL-6 levels	0.97	0.94-1.00	0.049

CI, confidence interval; HR, hazard ratio; ICI, immune checkpoint inhibitor; IL-6, interleukin-6.

*Defined as the combination of ipilimumab plus nivolumab.

Analyses were performed using a Fine and Gray competing risk regression model, considering immune-related adverse events as the main event and death as the competing event.

Factors included in the multivariate analysis were selected according to the directed acyclic graph diagram designed for the study (**Supplementary Figure S1**).

In accordance with the DAG diagram, in the multivariate Fine and Gray competing risk regression model including patient age, sex, pre-existing immune-mediated disease, dual ICI therapy and post-ICI IL-6 levels, only female sex (HR, 1.81; 95% CI, 1.17-2.81; p=0.008), dual ICI therapy (HR, 1.86; 95% CI, 1.14-3.02; p=0.012) and low post-ICI IL-6 levels (HR, 0.97; 95% CI, 0.94-1.00; p=0.049) remained independently associated with irAEs (**Table 3**). Furthermore, when post-ICI IL-6 levels were standardized, the effect seemed stronger, with an HR of 0.74 (95% CI, 0.55-1.00; p=0.049).

### 3.4 Subgroup analyses

**Figure 2:**
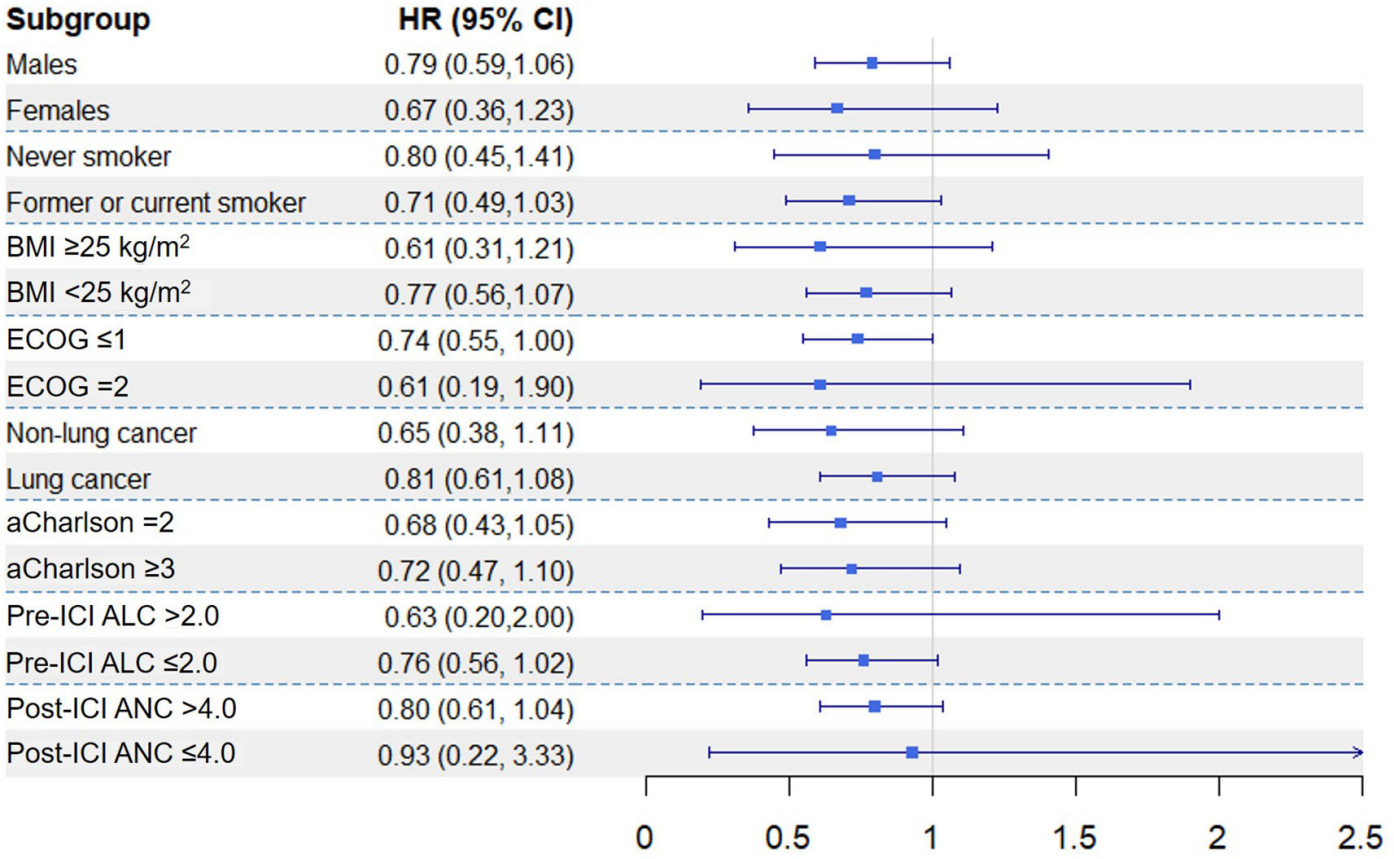
Adjusted hazard ratios for normalised interleukin-6 levels measured immediately before the second cycle of immune checkpoint inhibitors (post-ICI levels), presented in stratified subgroup analyses. Abbreviations (in alphabetical order): aCharlson, abbreviated Charlson comorbidity index; ALC, absolute lymphocyte count; ANC, absolute neutrophil count; BMI, body mass index; CI, confidence interval; ECOG, Eastern Cooperative Oncology Group; HR, hazard ratio; ICI, immune checkpoint inhibitor; IL-6, interleukin-6. Subgroup analyses were performed using Fine and Gray multivariate regression models, adjusted by age, sex, pre-existing immune-mediated disease and dual ICI therapy.

The impact of post-ICI IL-6 levels on irAEs did not vary by patient sex: the HR for normalized post-ICI IL-6 levels was 0.67 (95% CI, 0.36-1.23; p=0.190) in women and 0.79 (95% CI, 0.59-1.06; p=0.110) in men. Stratifying the cohort by the other prespecified categories, no differences were observed in the effect of post-ICI IL-6 levels on the cumulative incidence of irAEs across subgroups (**Figure 2**).

### 3.5 Incremental predictive value of interleukin-6 levels

The model including age, sex, pre-existing immune-mediated disease and dual ICI therapy yielded a Harrell’s C-index of 0.623. The inclusion of post-ICI IL-6 levels in this model improved the C-index to 0.640, representing an absolute increase of 0.017 in the model’s ability to classify patients according to their irAE risk.

## 4 Discussion

The main finding of this study is that low, rather than high, post-ICI IL-6 levels are associated with a higher risk of developing irAEs in cancer patients treated with ICIs. In addition to post-ICI IL-6 levels, other well-established risk factors, namely, female sex and dual ICI therapy, were also associated with a higher likelihood of irAEs in our cohort. The association between low post-ICI IL-6 levels and irAEs remained consistent across subgroups. Moreover, incorporating post-ICI IL-6 levels into an explanatory model of irAEs improved the model’s overall diagnostic performance.

The pathogenesis of irAEs is complex but, essentially, results from ICI-induced activation of the immune system, particularly of cytotoxic T cells, leading to autoimmune manifestations in cancer patients (33). By analogy with certain immune-mediated diseases that share similarities with irAEs (9), IL-6 has emerged as a promising biomarker for irAE diagnosis (34). In contrast to our results, many previous studies have associated an increased risk of irAEs with high IL-6 levels, both before and after ICI initiation (7, 21, 35). Notably, in most of these previous studies, IL-6 levels were measured at the time of irAE onset (21, 35). Taking a predictive approach that minimized the impact of frequent patient censoring on statistical power, we designed a prospective study in which IL-6 levels were measured before irAE onset and early during the course of ICI therapy. Furthermore, our study design allowed us to account for the dynamic effect of the first ICI dose, which is known to induce profound immunological changes (36, 37) and the influence of the post-ICI IL-6 levels on irAE incidence. The conflicting results reported across studies in this field may be attributed to variations in the timing of IL-6 measurements, which likely reflects the distinct clinical and immune status of patients at baseline, after the first ICI cycle and at irAE onset.

Patients with elevated circulating IL-6 levels are known to have a poorer response to ICI therapy and cancer-related prognosis than those with normal IL-6 levels (12, 38). Sustained IL-6 elevation promotes malignant cell immune evasion by modifying the tumor microenvironment (39). Specifically, IL-6 induces an immunosuppressive state via the JAK1/STAT3 pathway, which inhibits cytotoxic T-cell differentiation and anti-tumor activity (40). Consistently, in animal models of solid cancer, IL-6 receptor blockade enhances the anti-tumor efficacy of ICIs while mitigating certain autoimmune manifestations (41). In other words, when combined with ICI therapy, IL-6 antagonists may provide a synergistic anti-tumor effect in cancer patients without worsening toxicity.

Given that irAEs arise from ICI-induced T-cell activation, persistently elevated IL-6 levels, which are known to drive T-cell dysfunction, would be expected to act as a protective factor against irAE development. From a clinical perspective, this hypothesis is consistent with previous evidence demonstrating a strong association between the efficacy and toxicity of ICI therapy in cancer patients (5), as both are related to enhanced T-cell activity. In accordance with the tumor-immune contexture theory (42), IL-6 promotes the establishment of an immunologically dysregulated microenvironment that impairs the recruitment, expansion and activation of effector T cells (43), both locally and systemically (44, 45). By analogy with the low immunogenicity of ‘cold’ tumors (46), this IL-6-mediated immunosuppressive effect decreases the immunogenic potential of cancer cells, thereby reducing both patients’ responsiveness to ICIs and their susceptibility to irAEs (13). Accordingly, glucocorticoid-induced exogenous immunosuppression was also associated with poorer anti-tumor responses and lower rates of ICI-induced toxicities (47). Conversely, as we observed in our cohort, patients with pre-existing immune-mediated disease, particularly those with active disease, are at higher risk of developing irAEs than patients without such conditions (48, 49). The trend towards lower baseline IL-6 levels in our subgroup of patients with a history of immune-mediated disease further supports the association between irAEs and low post-ICI IL-6 levels, while also suggesting the involvement of an IL-6-independent pathway in the pathogenesis of these pre-existing diseases, such as the well-established IL-17 pathway in psoriasis. Another question is whether IL-6 levels may increase in the peripheral blood at the time of irAE onset, particularly in systemic or severe cases characterized by a hyperinflammatory response (50).

Although we did not find a significant interaction between female sex and post-ICI IL-6 levels, women in our cohort showed lower post-ICI IL-6 levels than men (data not shown), and consequently, were at a higher risk of developing irAEs. Notably, the observed association between female sex and increased risk of irAEs is supported by previous evidence (22), including a prospective study conducted by our group (23), and aligns with the well-documented susceptibility of women to autoimmune diseases compared to men. Moreover, in accordance with the findings of Valpione et al. (15), women remained at higher risk of irAEs than men for equivalent circulating IL-6 levels. Similar to peripheral neutrophil counts, which fluctuate between men and women over the years (51), physiological and pathogenic IL-6 levels associated with irAEs and other inflammatory conditions also differ depending on patient sex and age (24, 52). Consistent with the inflamm-aging theory (53), patients in our cohort aged 70 years or over had higher pre-ICI IL-6 levels than those under 70. However, in contrast to a previous pilot study by our group, in which younger patients were observed to be predisposed to irAEs (54), age was not associated with irAE incidence in the current analysis. This lack of association between irAEs and age, which reflects the conflicting findings in the literature (55), may be attributable to other factors, such as ECOG performance status and aCharlson comorbidity index, in which older patients scored worse and both correlated with higher IL-6 levels in our cohort.

Regarding the ICI regimen administered, the combined treatment with ipilimumab and nivolumab is a well-established risk factor for irAE incidence and severity (1), with ICI-induced IL-6 release being one of the proposed underlying mechanisms (56). The high toxicity profile associated with dual ICI therapy highlights the need for proactive strategies to prevent irAEs, including biomarker-guided interventions. In this context, the combination of ICI therapy with tocilizumab or sarilumab, two monoclonal antibodies targeting the IL-6 receptor, is currently under investigation by ongoing clinical trials as a potential pre-emptive strategy for ICI-induced toxicities (NCT04940299, NCT05428007, NCT03999749). The rationale for the prophylactic use of anti-IL-6 receptor antibodies in combination with ICIs is supported by the favorable safety profile of IL-6 blockade in terms of cancer-related outcomes (19). Notably, in patients receiving chimeric antigen receptor T (CAR-T) cells, the administration of tocilizumab to mitigate cytokine release syndrome does not adversely affect the progression of the underlying hematological malignancy (57). Moreover, tocilizumab has been successfully used to prevent cytokine release syndrome in patients with non-Hodgkin’s lymphoma just before CAR-T cell infusion, with excellent results in terms of toxicity and antitumor activity (58). Nevertheless, the main association observed in our study -between low post-ICI IL-6 levels and risk of irAEs- underscores the need to reconsider the design of future clinical trials exploring the benefit of IL-6 blockade in combination with ICIs for irAE prevention.

The current study has several limitations that should be acknowledged. First, the frequency of certain irAE types, such as neurological toxicities, was lower than previously reported (59). This discrepancy may in part be attributed to the study design, which focused on predictive biomarkers (i.e., factors present before irAE onset) and considered only the first irAE for each patient. Nevertheless, although not included in the statistical analysis, we identified neurological irAEs occurring beyond the first irAE during the follow-up (data not shown). In addition, severe irAEs were underrepresented, most events in this study being grade 1 or 2. From a clinical perspective, however, early detection of less severe irAEs may be valuable, as it is widely accepted that mild irAEs may precede the onset of higher-grade or multisystem toxicities (31). Second, although estimated, the sample size of the cohort was small, as reflected in the wide confidence intervals in the subgroup analyses. Third, this study focused on IL-6 levels, which are available in clinical practice, but did not take into account other biomarkers potentially involved in irAE pathogenesis. A broader laboratory analysis with additional cytokines and chemokines could further improve irAE risk classification and refine our results. There was some missing information in the dataset, specifically regarding post-ICI IL-6 levels, which could have influenced the results. However, given the small proportion of missing data and the consistency observed between the univariate analyses (performed with all available data) and the multivariate analyses (restricted to complete cases), any potential impact is likely to be minimal. Finally, due to high cancer-related mortality, the follow-up period was relatively short for some patients and varied across the cohort. This time-related heterogeneity was addressed using Fine and Gray competing risk models, which accounted for irAE risk while considering death as a competing event. Despite these limitations, this study, which is based on a real-world multicenter prospective pan-cancer cohort specifically designed to identify irAE risk factors, provides valuable insights into ICI-related toxicity.

## 5 Conclusion

Besides other well-established risk factors such as female sex and dual ICI therapy, low rather than high post-ICI IL-6 levels were associated with irAE occurrence in a pan-cancer cohort of patients treated with ICIs. The addition of post-ICI IL-6 levels to a model including other explanatory factors improved (modestly) the overall diagnostic performance. Despite the potential clinical utility of IL-6 levels in cancer patients receiving ICIs, there is a need for new reliable validated biomarkers for irAE prediction.

## Data Availability

The raw data supporting the conclusions of this article will be made available by the authors, without undue reservation.
