# Supplementary material for: Low rather than high interleukin-6 levels are associated with immune-related adverse events in cancer patients treated with immune checkpoint inhibitors
Source: Front Immunol. 2025 Sep 12;16:1677778. doi: 10.3389/fimmu.2025.1677778 (PMC12464006; doi:10.3389/fimmu.2025.1677778)
Supplement: Supplementary file 1 [file SupplementaryFile1.docx]

Supplementary Material

**Supplementary Figure 1.** Directed acyclic graph diagram of the study.


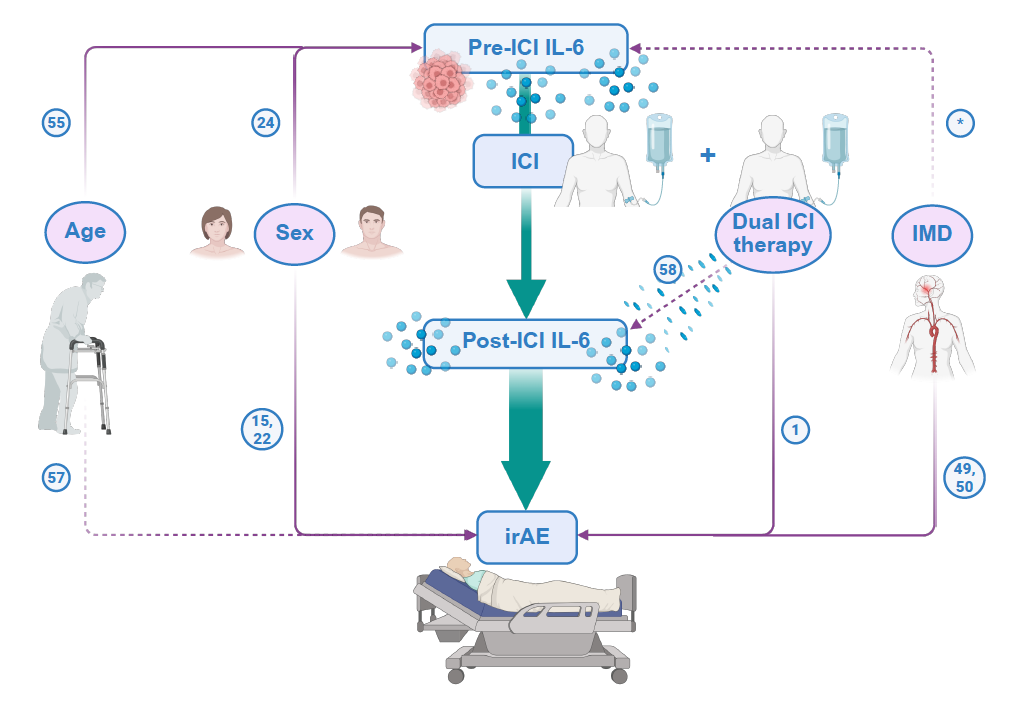


**Abbreviations (in alphabetical order):** ICI, immune checkpoint inhibitor; IL-6, interleukin-6; IMD, immune-mediated disease; irAE, immune-related adverse event.

Solid green arrows indicate associations under investigation in this study.

Solid purple arrows indicate well-established associations supported by prior evidence.

Dashed purple arrows indicate associations that remain controversial despite previous findings.

Numbers in circles correspond to reference numbers cited in the manuscript that support each association.

* Baseline IL-6 levels may vary depending on the specific pre-existing immune-mediated disease.

Figure created with BioRender.com.

**Description:** The study hypothesis was that pre- and post-ICI IL-6 levels may be associated with irAE incidence in cancer patients receiving ICIs (solid green arrows). Accordingly, pre- and post-ICI IL-6 levels were evaluated as exposure variables, while other potential irAE risk factors, namely, patient age, sex, pre-existing immune-mediated disease and dual ICI therapy, were considered confounders. Female sex, pre-existing immune-mediated disease and dual ICI therapy are well-established risk factors for irAEs (solid purple arrows at the bottom), while the association between age and irAE occurrence remains controversial (dashed purple arrow at the bottom). Although not specifically investigated in cancer patients, age and sex are known to influence baseline IL-6 levels (solid purple arrows at the top), which may, in turn, vary depending on the specific pre-existing immune-mediated disease (dashed purple arrow at the top). Some studies have reported a significant increase in IL-6 levels associated with dual ICI therapy, even after the first cycle (dashed purple arrow near the center of the figure).

**Supplementary Figure 2.** Interleukin-6 levels at baseline (pre-ICI sample) and immediately before the second ICI cycle (post-ICI sample) in the 207 patients who reached the second ICI cycle.

**Abbreviations (in alphabetical order):** CI, confidence interval; ICI, immune checkpoint inhibitor; IL-6, interleukin-6.

Pre- and post-ICI IL-6 levels were compared using a Wilcoxon signed-rank test for paired data.

**Supplementary Figure 3.** Cumulative incidence of immune-related adverse events over time in the presence of death as a competing risk.


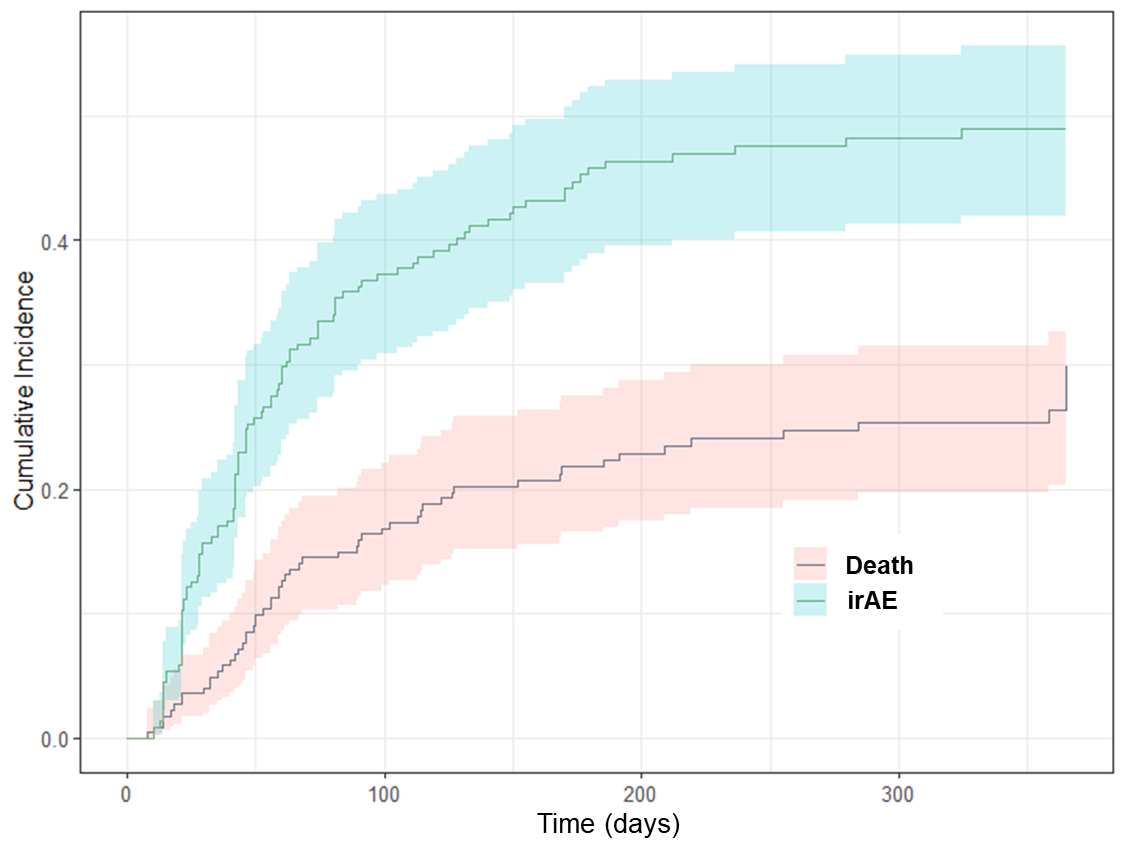


**Abbreviations:** irAE, immune-related adverse events.

The plot was generated using the Fine and Gray competing risk survival analysis, considering immune-related adverse events as the main event and death as the competing event. The colors show the 95% confidence interval.

**Supplementary Table 1.** Baseline Interleukin-6 Levels in Cohort Patients Stratified by Demographic and Clinical Characteristics, and Use of ICI Therapy with Adjuvant Intent.

|  | n | IL-6 levels  (pg/ml) | *p*-value |
| --- | --- | --- | --- |
| *Age* |  |  |  |
| < 70 years | 134 | 4.87±13.86 | 0.001 |
| ≥ 70 years | 90 | 9.07±17.04 |  |
| *Sex* |  |  |  |
| Female | 56 | 4.95±8.71 | 0.163 |
| Male | 168 | 7.09±16.95 |  |
| *ECOG score* |  |  |  |
| 0 | 65 | 2.13±4.23 | < 0.001 |
| 1-2 | 159 | 8.37±17.69 |  |
| *Smoking history* |  |  |  |
| Never smoker | 46 | 6.02±9.53 | 0.755 |
| Former or current smoker | 178 | 6.70±16.51 |  |
| *Body mass index* |  |  |  |
| < 25 kg/m^2^ | 109 | 7.91±19.44 | 0.655 |
| ≥ 25 kg/m^2^ | 115 | 5.28±9.89 |  |
| *Chronic renal failure** |  |  |  |
| No | 179 | 6.08±13.93 | 0.602 |
| Yes | 45 | 8.47±19.99 |  |
| *Pre-existing immune-mediated disease* |  |  |  |
| No | 207 | 6.91±17.87 | 0.174 |
| Yes | 17 | 2.28±2.55 |  |
| *Abbreviated Charlson index* |  |  |  |
| 2 | 120 | 5.41±14.08 | 0.003 |
| 3 or higher | 104 | 7.89±16.61 |  |
| *Primary cancer origin* |  |  |  |
| Lung cancer | 84 | 8.94±18.64 | 0.008 |
| Non-lung cancer | 140 | 5.13±12.79 |  |
| *Use of ICI therapy with adjuvant intent* |  |  |  |
| Adjuvant | 29 | 1.20±1.14 | < 0.001 |
| Non-adjuvant | 195 | 7.36±16.27 |  |

**Abbreviations (in alphabetical order):** ECOG, Eastern Cooperative Oncology Group; ICI, immune checkpoint inhibitor; IL-6, interleukin-6; n, number.

* Defined as a baseline glomerular filtration rate below 60 mL/min/m^2^.

Interleukin-6 levels are expressed as mean ± standard deviation.

Comparisons between groups were made using a non-parametric Mann-Whitney *U* test for unpaired data.
